# Brown Adipose Tissue Is Associated with Reduced Weight loss and Risk of Cancer Cachexia: A Retrospective Cohort Study

**DOI:** 10.1016/j.clnu.2024.12.028

**Published:** 2024-12-30

**Keywords:** obesity, cancer, muscle, cachexia, brown adipose tissue

## Abstract

**Background & Aims:**

Brown adipose tissue (BAT) has been mainly investigated as a potential target against cardiometabolic disease, but it has also been linked to cancer-related outcomes. Although preclinical data support that BAT and the thermogenic adipocytes in white adipose tissue may play an adverse role in the pathogenesis of cancer cachexia, results from studies in patients have reported inconsistent results. The purpose of this study was to examine the interrelationship between presence of detectable BAT, changes in body weight, and cachexia in patients with cancer. We hypothesized that evidence of BAT at cancer diagnosis would be associated with greater weight loss and risk of cancer cachexia up to a year after cancer diagnosis.

**Methods:**

We conducted a retrospective cohort study in treatment-naïve patients with detectable BAT (BAT+, n = 57) and without evidence of BAT (BAT-, n = 73) on 2-deoxy-2-[^18^F]fluoro-D-glucose positron emission tomography-computed tomography (^18^F-FDG-PET-CT) imaging performed for cancer staging (2004-2020). Patients’ clinical, demographic, and anthropometric characteristics were extracted from their electronic medical record for up to a year after diagnosis. The two groups were *a priori* matched for demographic, anthropometric, and disease-related characteristics at diagnosis, as well as for season and outdoor temperature on the day of the PET-CT scan. Cancer cachexia was defined as weight loss greater than 5% or 2% if body mass index was lower than 20 kg/m^2^. Poisson regression models were fitted to estimate the relative risk (RR) for developing cancer cachexia over the 1-year follow-up among BAT+ compared to BAT- patients.

**Results:**

The BAT+ group experienced a lower magnitude of weight loss compared with the BAT- group during the 1-year follow-up (p = 0.014 for interaction between BAT status and time). The risk for cancer cachexia was 44% lower in the BAT+ than the BAT- group, adjusted for age, sex, outdoor temperature on the day of the ^18^F-FDG-PET-CT) imaging, cancer site and stage (RR: 0.56, 95% CI: 0.32 to 0.97).

**Conclusion:**

Contrary to our initial hypothesis, evidence of BAT assessed by ^18^F-FDG-PET-CT imaging at cancer diagnosis was associated with greater body weight maintenance and lower risk for developing cancer cachexia up to one year after diagnosis. Larger, prospective studies and mechanistic experiments are needed to expand and identify the causal factors of our observations.

## Introduction

Cancer cachexia is a multifactorial and sometimes irreversible syndrome characterized by pronounced unintentional weight loss and skeletal muscle wasting [1] impacting cardiorespiratory function, quality of life, and treatment effectiveness as well as the survival of patients with cancer [2–5]. Cancer cachexia affects approximately 50-80% of cancer patients (depending on the tumor type) and is thought to contribute to 22-30% of cancer-related deaths [6, 7]. The mechanisms underlying the development of cancer cachexia have not been clearly elucidated, but are thought to involve a range of tumor-derived inflammatory and immune-related mediators that trigger inflammation, proteolysis, and lipolysis [8, 9] leading to loss of skeletal muscle and adipose tissue mass. Although some therapeutic options have been suggested for the management of cancer cachexia (e.g., nutrition counseling, progesterone analogs, androgens, and corticosteroids among others), their effectiveness is modest [10]. More recently, inhibition of growth differentiation factor 15 (GDF-15) has been proposed as a potential therapeutic approach with encouraging results in patients with cancer cachexia [11].

Brown adipose tissue (BAT) and the “browning” of the white adipose tissue (WAT) - i.e., the emergence of brown-like adipocytes in classical WAT depots - has attracted research interest due to its high capacity for nutrient utilization to produce heat, a wasting process [12]. To this date, BAT has been mostly studied in the context of obesity and its related metabolic perturbations suggesting that increased BAT volume is associated with improved metabolic function and lower probability of having a diagnosis of type 2 diabetes, cardiovascular diseases, and hepatic steatosis [13–17]. However, BAT and the thermogenic adipocytes in WAT have been implicated in the pathophysiology of various wasting conditions (e.g., cancer cachexia, burn injury and pheochromocytoma) [18]. Although preclinical data support that BAT and the browning of WAT may contribute to the pathogenesis of cancer cachexia [19–23], results from studies in patients have reported inconsistent results [24–28].

The purpose of this study was to examine the interrelationships between BAT, changes in body weight, and cachexia in patients with cancer. To address this question, we conducted a retrospective cohort study in adult patients with diagnosed primary malignancies of various origins. We hypothesized that patients with detectable BAT in their diagnostic 2-deoxy-2-[^18^F]-fluoro-glucose (^18^F-FDG) positron emission tomography-computed tomography (PET-CT) imaging scan would exhibit more pronounced weight loss and have higher risk of developing cancer cachexia up to 12 months after diagnosis compared to patients without detectable BAT.

## Material and Methods

### Patient population

To identify patients with cancer and evidence of active BAT, we retrospectively queried the radiological reports database of all patients who had undergone ^18^F-FDG-PET-CT for cancer diagnosis and/or staging at University of California Davis (UC Davis) from December 2004 until July 2020, for evidence of detectable BAT depots (BAT+). Subsequently, we queried the electronic medical records to identify patients with newly diagnosed cancer who also underwent staging ^18^F-FDG-PET-CT with no evidence of BAT presence (BAT-) in their radiologic report. The inter-reader variability for the assessment of BAT has been previously reported to be very high using ^18^F-FDG-PET-CT imaging [29]. Eligible participants had to be at least 18 years old, recorded a staging ^18^F-FDG-PET-CT prior to cancer-specific treatment, and provided electronic medical records for at least 6 months following the initial cancer diagnosis. Exclusion criteria included prior cancer diagnosis/treatment within 5 years, use of medications that may affect BAT activity (i.e., alpha or beta- adrenergic blockers or agonists and steroids), incomplete medical records (missing height and weight data at diagnosis), pregnancy, and prisoners. Based on these criteria, we identified a group of BAT+ patients (n = 75) and a group of BAT- (n = 257). To minimize the effect of confounding factors on the relationship of the BAT presence or absence with weight loss and cancer cachexia, out of the previously identified BAT+ and BAT- groups, we selected a group of BAT+ patients (n = 57) who were matched with a group of BAT- patients (n = 73) with similar age, sex, body mass index (BMI), race, ethnicity, outdoor temperature during the day of the ^18^F-FDG-PET-CT imaging session, cancer type and grade. This study was approved by the UC Davis Institutional Review Board in accordance with the Declaration of Helsinki.

## Power calculation

Based on the current literature [30], patients with cancer experience (mean ± SD) 7.0% ± 6.7% body weight loss 6 months after diagnosis. Assuming the same variability, we estimated that 48 patients would be minimally needed in each group to detect a 5% difference in weight loss trajectory since cancer diagnosis (primary outcome) between the two groups (which is realistic and clinically meaningful [31]) with >95% power using two-sided tests at the α = 0.05 level of significance.

### Cancer Diagnosis and Staging

Primary cancer sites were classified to one of the eight categories: 1) hematologic (all lymphoma types, multiple myeloma, large B-cell lymphoma, chronic myeloid leukemia); 2) genitourinary (endometrial, cervix, renal cell, urothelial, squamous cell carcinoma of the bladder); 3) skin (carcinomas, melanoma, squamous cell carcinoma with no primary origin noted, Merkel cell carcinoma, basosquamous carcinoma, large cell carcinoma); 4) respiratory (lung-based tumours, mucinous adenocarcinoma, adenosquamous carcinoma, small cell carcinoma of the lung, squamous cell carcinoma of the lung, clear cell carcinoma of the lung, adenocarcinoma without a tissue diagnosis, non-small cell lung carcinoma, thymoma); 5) gastrointestinal (cholangiocarcinoma, colorectal, intestine neuroendocrine); 6) breast (lobular carcinoma, any breast tissue malignancy, in situ/ invasive ductal carcinoma); 7) head and neck (squamous cell carcinoma with origin in the head or neck region, basaloid squamous cell carcinoma, adenoid cystic carcinoma); and 8) other cancer types of various origins (neurofibroma, paraganglioma, myxoid liposarcoma, leiomyosarcoma, fibromyxosarcoma, malignant neoplasm of unknown primary). The cancer stage for solid tumours was decided using the Tumour, Node, and Metastasis (TNM) staging system, categorizing it into four stages (I–IV) and was obtained from the CNEXT registry at UC Davis [32].

### Definition of Cancer Cachexia

Cancer cachexia was determined in accordance with the international consensus on the definition of cachexia: i) weight loss greater than 5% or ii) weight loss greater than 2% if BMI is lower than 20 kg/m^2^ [31].

### Other Characteristics

Patients’ clinical and histological data (cancer type, stage, and grade), demographic (age, gender, race, and ethnicity), and anthropometric characteristics (height and weight) of the were extracted from their electronic medical records during the first year after diagnosis. The local average, minimum and maximum temperatures of the day of the scan were extracted for the historic temperature dataset available via the National Oceanic and Atmospheric Administration [33].

### ^18^F-FDG-PET-CT imaging

^18^F-FDG-PET-CT scans were performed using a Discovery ST or 690 scanners (General Electric, Boston, MA). The patient preparation protocol included fasting for 4-6 hours and avoiding strenuous exercise for at least 24 hours prior to the imaging visit. Patients received either 740 MBq (on Discovery ST) or 370 MBq (on Discovery 690) and the ^18^F-FDG-PET-CT imaging was performed (mean and standard deviation (SD)) 71 ± 12 minutes later. According to the clinical imaging protocol, the patients first performed a CT scan (140 kVp, 300 mAs) followed by a PET scan (3-4 minutes per bed position depending on the patient’s size) from skull base to mid-thigh, or from vertex to toes depending on the clinical indication. The CT was reconstructed with filtered back projection into a 512 x 512 matrix resulting in voxel size of 0.98 x 0.98 x 3.75 mm. For the Discovery 690 scanner, PET images were reconstructed into a 192 x 192 matrix using an ordered subsets expectation maximization (OSEM) algorithm with two iterations and 24 subsets and incorporating time-of-flight (TOF) and all standard corrections (attenuation, scatter, randoms, deadtime, and normalization). No point spread function modeling was employed. A post-reconstruction Gaussian smoothing was applied with 6.4 mm full width at half maximum (FWHM). The resulting PET voxel size was of 3.65 x 3.65 x 3.27 mm. On Discovery ST scanner, PET images were reconstructed into 128 x 128 matrix using OSEM algorithm with 2 iterations and 30 subsets. The resulting voxel size was 5.47 x 5.47 x 3.27 mm.

### PET-CT image analysis

**Figure 2:**
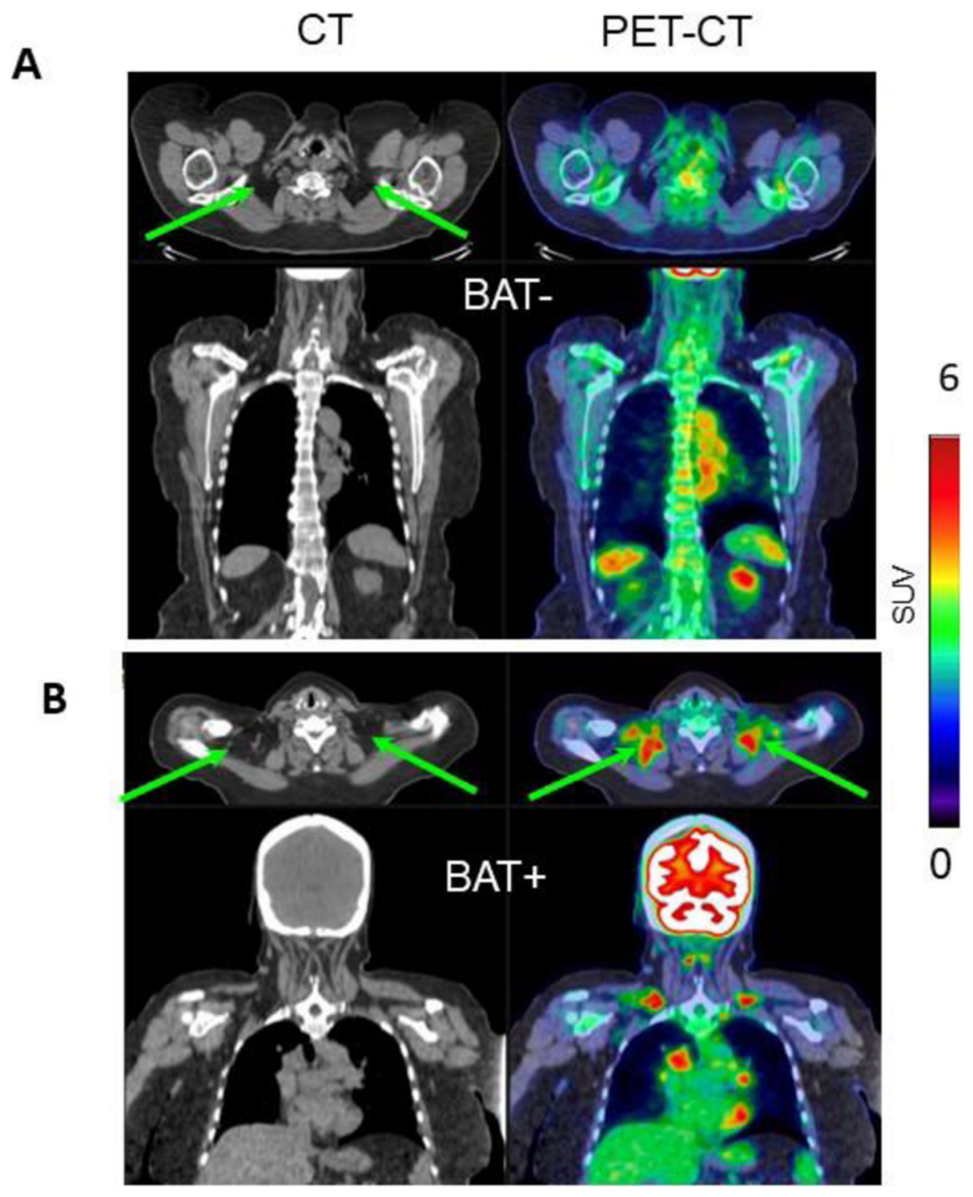
BAT assessment using ^18^F^-^FDG-PET-CT imaging. Representative images - axial (top) and coronal (bottom) images - from **(A)** a patient in the BAT- and **(B)** the BAT+ group. The supraclavicular adipose tissue depot is highlighted with green arrows on the CT component and the fused image of the study. *Abbreviations: BAT, brown adipose tissue; BAT+, patient with detectable BAT; BAT-, patient without detectable BAT;* ^*18*^*F*^*-*^*FDG, 2-deoxy-2-[18F]fluoro-D-glucose; PET, Positron Emission Tomography; CT, computed tomography*.

BAT volume and activity were quantified in the BAT+ group according to BARCIST recommendations [34]. The analysis of PET-CT imaging was conducted using Life X software version 6.2 [35]. ^18^F-FDG uptake was quantified as a standardized uptake value (SUV) normalized to the individual’s estimated lean body mass (LBM) which was calculated using the Janmahasatian formula [36]. BAT volume and activity were quantified using a threshold corrected for LBM, SUV_LBM_ = (1.2 g/mL)/(LBM%) [34]. After ensuring adequate registration between the CT and PET components of each scan, a threshold-based method was applied to quantify BAT metrics. Specifically, a large initial volume of interest (VOI) was placed on CT slices between C3 and T3 vertebrae; to capture the neck and paravertebral brown fat depots. Then, a CT-based contour that included radiodensity within the -190 to -10 Hounsfield Units was defined [34]. A new threshold was added on the PET component based on the SUV above the individually determined SUV_LBM_ cut-off. The intersected contour that fulfilled the two threshold conditions (on CT and PET) was used to derive BAT metrics including active BAT volume. Before deriving any metrics, the contour was reviewed slice-by-slice to avoid including other tissues with high ^18^F-FDG uptake either pathologic (e.g., tumour foci) or physiologic (e.g. soft palate, esophagus, bone marrow, muscles, or myocardium) (Figure 2).

### Statistical analysis

Analyses were performed using the following software SPSS (IBM Corp. Version 26.0, Armonk, NY), Stata Statistical Software version 17.0 (College Station, TX: StataCorp LLC) and Prism (GraphPad, Boston, MA). Data are presented as mean ± SD or 95% confidence interval (CI) for normally distributed variables or median (interquartile range, IQR) for non-normally distributed parameters. Categorical variables are presented as absolute number of observations and percentages (%). Crude differences between BAT+ and BAT- groups in patient characteristics were assessed using Students t-test for normally distributed data and the Mann-Whitney U test for skewed data. Chi-squared or Fisher’s Exact test was used to analyze differences in categorical variables (such as sex, race, ethnicity) between the study groups. For hypothesis testing, a mixed-effects analysis was employed to examine the between-group differences for changes in body weight over time, with a subsequent post-hoc Sidak’s procedure for pairwise-comparisons across different study time points. For the association of the BAT status with the risk of developing cancer cachexia, a repeated-measures Poisson regression was fitted to estimate the relative risk (RR) and 95% CI with statistical adjustments for potential covariates. The level of statistical significance was set at 0.05 for all analyses.

## Results

### Patient population

Table 1 contains the demographic, clinical, anthropometric, and other relevant information of the patients included in the BAT+ and BAT- groups. By study design, there were no significant differences between groups in any matching factors: demographic, anthropometric, clinical, or cancer-related characteristics (all comparisons: p > 0.05). Similarly, we observed no statistically significant differences in the temperature (minimum, maximum, and average) on the day of the scan and season that PET-CT scans took place. For the BAT+ group, the median BAT volume was 38.0 mL (IQR: 16.9 to 83.1 mL) with median SUV_mean_ of 2.4 g/mL (IQR: 2.2 to 2.8 g/mL) and median SUV_max_ 5.9 g/mL (IQR: 4.2 to 8.5 g/mL). The mean BAT radiodensity was -57.2 ± 10.2 Hounsfield units. The BAT- group had no detectable BAT according to relevant radiological report and after visual inspection by our research team.

### Changes in body weight after cancer diagnosis in patients with and without detectable BAT

**Figure 3:**
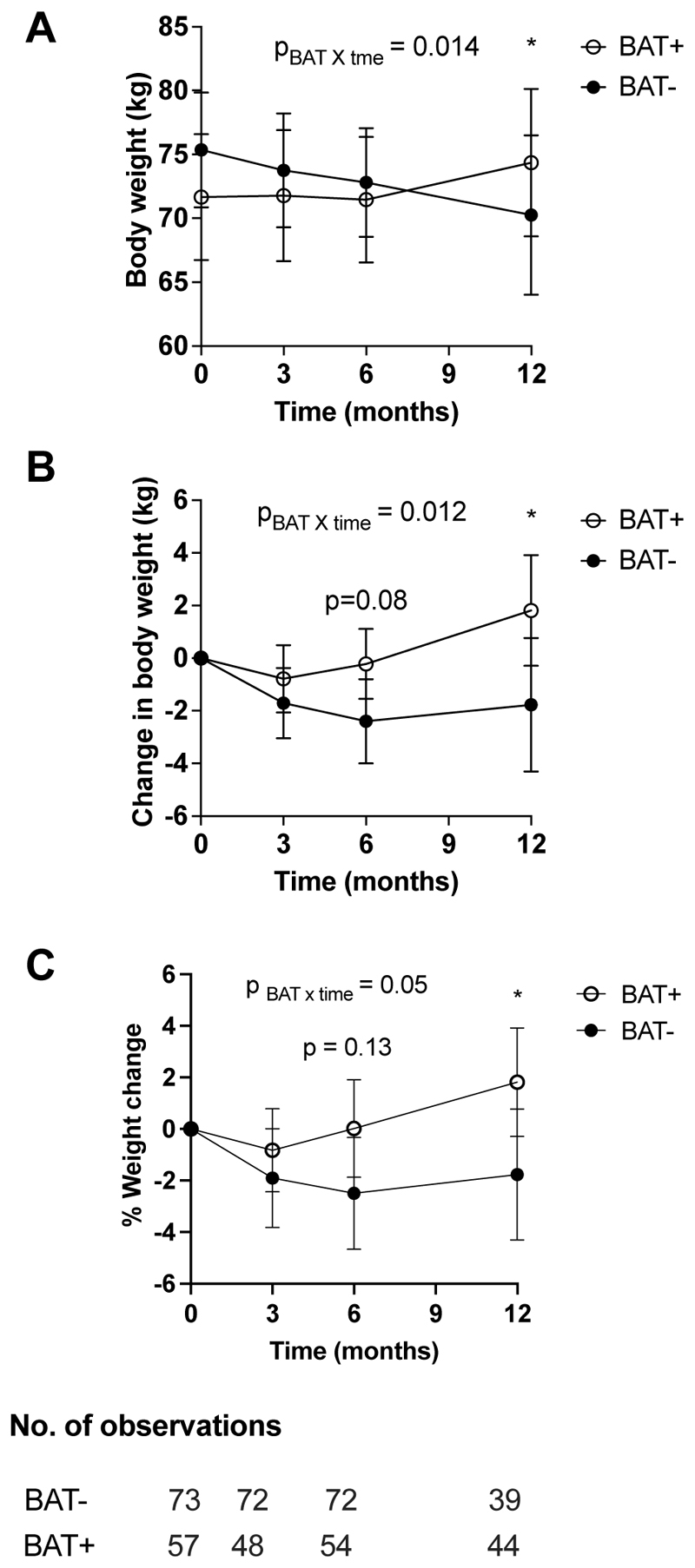
Body weight (A), absolute weight change (B), and relative weight change (C) trajectories by BAT status up to 12 months after cancer diagnosis. P-values for interaction were calculated using mixed effects analysis and significant interactions were followed by Sidak’s post hoc procedure. Data are mean and 95% confidence interval. *p<0.05. *Abbreviations: BAT, brown adipose tissue; BAT+, patients with detectable BAT; BAT-, patients without detectable BAT*. Body weight parameters were not available for one patient of the BAT- group up to 6 months and 34 patients at 12 months, while such data were missing for three patients of the BAT+ group at 6 months and 13 patients at 12 months due to lack of such measurements in the respective visits, transfer to a different health care institution or death.

We found a significant interaction between BAT group and time for body weight (p-value for interaction = 0.014, Figure 3A), absolute weight change (p-value for interaction = 0.012, Figure 3B) and relative weight change from cancer diagnosis (p-value for interaction = 0.051, Figure 3C) between the two groups and across the study timepoints. The BAT+ group exhibited a relatively stable body average weight (change in weight at 6 months: -0.2 kg; 95% CI: -1.5 to 1.1 kg or 0.2%; 95% CI: -1.8 to 1.9%) up to 6 months after diagnosis with a trend for small weight gain at 12 months (change in weight at 12 months: 1.8 kg; 95% CI: -0.3 to 3.9 kg or 1.8%; 95% CI: -0.3 to 3.9%) after diagnosis. Conversely, the BAT- group demonstrated progressive weight loss (change in weight at 6 months: -2.4; 95% CI: -3.9 to -0.8 kg or -2.5%; 95% CI: -4.7 to -0.3% and 12 months: -1.7 kg; 95% CI: -4.2 to 0.7 kg or -1.8%; 95% CI: -4.3 to 0.8%) with significant post-hoc difference between groups at 12 months (p<0.05 for both absolute weight and weight change, Figure 3B-C).

### Comparison of the risk for developing cancer cachexia in patients with and without detectable BAT

**Figure 4:**
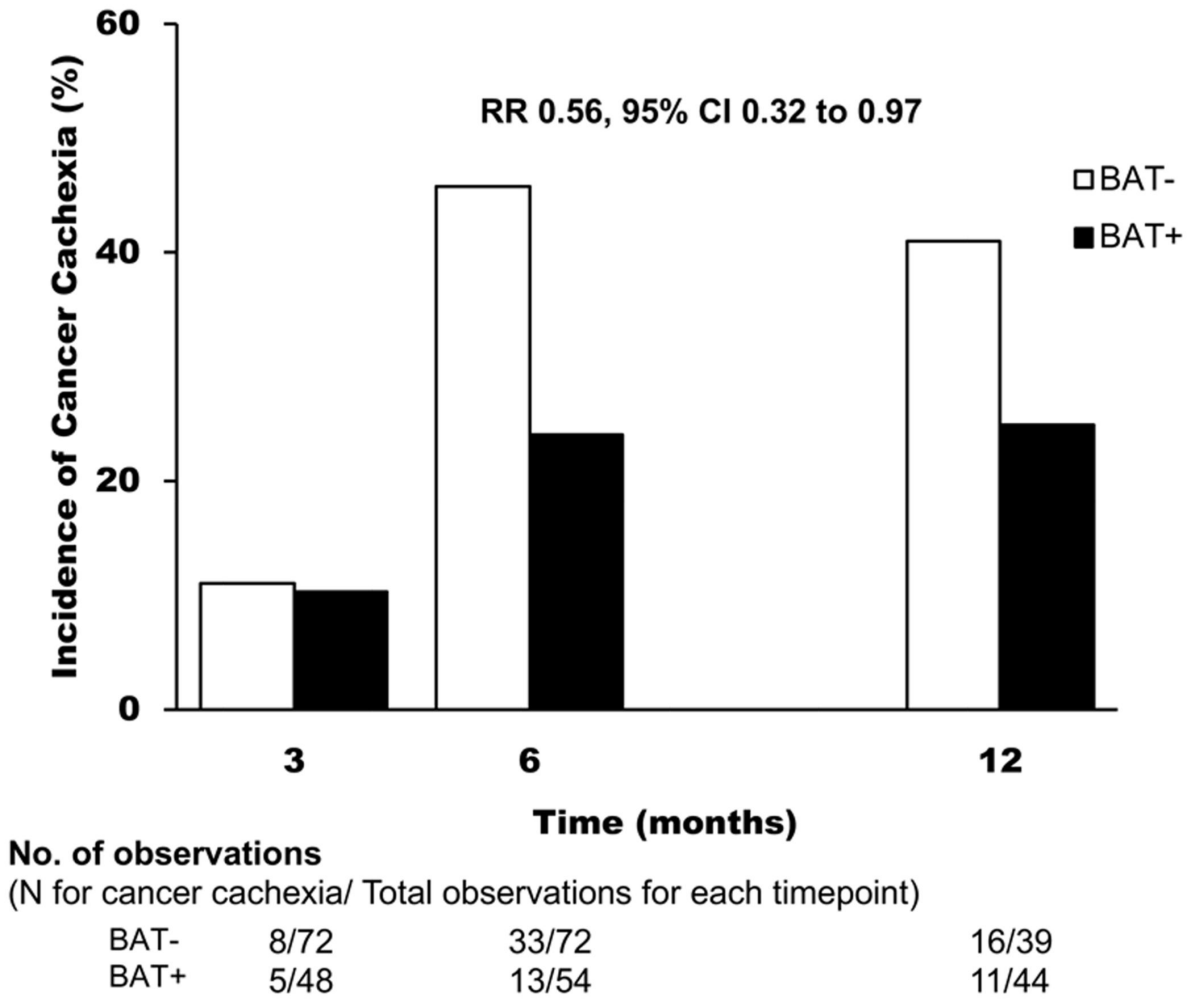
Incidence of cancer cachexia up to 12 months after cancer diagnosis by BAT status. Relative risk (RR) of the BAT+ group compared to the BAT- group for developing cancer cachexia across study timepoints was estimated using a repeated-measured Poisson regression model adjusted for age, sex, mean outdoor temperature at the day of the PET/CT, cancer site and stage. *Abbreviations: BAT, brown adipose tissue; BAT+, patients with detectable brown adipose tissue; BAT-, patients without detectable brown adipose tissue*.

Applying the definition of cachexia [31] to patients’ weight status, we found that in the BAT- group, eight patients developed cancer cachexia at 3 months (11%), 33 patients (46%) at six months and 16 patients (41%) at 12 months (Figure 4). In the BAT+ group, cachexia was present in five patients (10%) at 3 months, 13 patients (24%) at six months and 11 patients (25%) at 12 months, respectively. The overall incidence of cancer cachexia anytime in the first 12 months after diagnosis was 46.6% and 66.1% in the BAT+ and BAT- groups (p = 0.03), respectively. In the unadjusted model, the average risk over the 1-year follow-up was lower by 43% (RR 0.57, 95% CI: 0.36 to 0.85) in patients of the BAT+ group compared to the BAT- group. Adjustment for age, sex, mean outdoor temperature on the day of the PET/CT, and cancer site and stage did not materially affect these results indicating that the average risk over the 1-year follow-up was lower by 44% (RR: 0.56, 95% CI: 0.32 to 0.97) in patients of the BAT+ group compared to the BAT- group.

## Discussion

Cachexia is a wasting syndrome affecting a large proportion of patients with cancer as well as impacting their quality of life and survival [2–5]. The mechanisms underlying the development of cancer cachexia have not been clearly elucidated and no approved effective treatment approaches are currently available against it. Studies in preclinical models have implicated BAT and the browning of WAT in the pathogenesis of cancer cachexia. However, results from studies in people have elicited mixed results. Using a retrospective cohort study design, we found that treatment-naive patients with evidence of BAT on ^18^F-FDG-PET-CT imaging at cancer diagnosis experienced lower weight loss and risk for cachexia compared to a matched group of patients without evidence of BAT at diagnosis.

The reported protective relationship between BAT at diagnosis and risk of developing cancer cachexia contrasts our initial hypothesis that was based on the published preclinical and preliminary clinical data on the role of the thermogenic adipocytes in cancer cachexia. Specifically, BAT thermogenesis and the browning of WAT have been thought to contribute to the hypermetabolic state of cancer cachexia in rodents [19–23]. Further, pharmacologic and genetic manipulations affecting inflammation and the sympathetic nervous system have been reported to inhibit the browning of WAT and to ameliorate cancer cachexia in mice [21, 23]. Conversely, cold-induced BAT activation in rodents has been reported to inhibit tumor growth by lowering glucose availability in the tumor microenvironment [37]. To this date, only a small number of observational studies have explored the potential role of BAT in cancer-related outcomes yielding mixed results. The first report on the link between BAT and cancer cachexia in humans was a necropsy study suggesting higher prevalence of multilocular adipocytes - resembling brown adipocytes - in the perirenal adipose tissue depot of patients who died from cancer compared to patients with other cause of death [24]. However, the two groups were matched for age but not for sex and anthropometric characteristics. More recently, a larger retrospective study including two separate cohorts (n_1_ = 460 patients with active BAT and n_2_ = 283 patients with active BAT and at least two scans) suggested that BAT activity is not associated with any changes in the calculated cancer burden, used as a marker of disease activity and grading system, between consecutive scans [26]. However, in the latter study changes in body weight and/or weight loss as well as BAT volume were not quantified. Moreover, Eljalby et al. reported a trend for lower prevalence of cancer cachexia (defined similarly to the present study, in accordance with validated international consensus criteria [31]) in patients with detectable BAT than those without, after adjusting for potential confounders, but their results did not reach statistical significance [27]. Our study suggests that evidence of detectable BAT at cancer diagnosis may be protective for developing cachexia and thus this needs to be tested with prospective and mechanistic studies in the future.

The adipose tissue is now recognized as a heterogeneous endocrine organ involved in the metabolic regulation of several (patho)physiological processes [38]. Adiposity and its related metabolic complications (i.e., insulin resistance, type 2 diabetes) have been associated with adverse cancer-related outcomes including mortality, cancer recurrence and cachexia [39–42] and treatment of obesity and its associated conditions could be an effective approach to improve cancer incidence and cancer-related outcomes [43–45]. An increased BAT volume/activity in people has been linked to improved metabolic function and lower odds of type 2 diabetes, coronary heart disease, congestive heart failure and dyslipidemia among others [13–17], likely due to its increased capacity for substrate oxidation and/or its potential secretory role affecting metabolic function in other tissues [18, 46]. Considering the current knowledge in human BAT and its role in metabolism, the results of the current study suggest that individuals with detectable BAT, who are also likely to be metabolically healthier compared to those with no detectable BAT, can endure the clinical sequelae of cancer without developing cancer cachexia. Alternatively, it is possible that increased BAT activity in people with detectable BAT may decrease substrate availability to the tumour microenvironment leading to decreased tumor growth and that could potentially translate to other improved cancer related outcomes including cancer cachexia and/or there is a direct anti-cachectic, endocrine signal derived from BAT tissue.

Our study has strengths and limitations that need to be considered when interpreting its results. We have collected and analyzed longitudinal data at different timepoints for up to a year post cancer diagnosis and were able to show the trajectory in body weight and weight loss over time. The detailed characterization of our patients as well as the matched groups of patients with and without detectable BAT for several variables, including cancer related parameters and the outdoor temperature on the day of the imaging should be listed among its strengths. Although the two groups were matched for cancer site and stage, small, non-statistically significant differences in these variables may have introduced residual confounding in the relationship between BAT status and study outcomes. Nevertheless, the association between BAT and the risk of developing cancer cachexia remained largely unaffected after adjusting for cancer site, stage, and other potential confounders. Additional limitations of the study include its retrospective nature and the small sample size; nevertheless, the sample size was adequately powered to address the primary outcome of this investigation. Although loss of muscle mass constitutes one of the three diagnostic criteria for cancer cachexia [31], information on patients’ muscle mass was not available in the electronic medical records. We appreciate that depending on the histology of the cancer, the TNM staging system alone may not provide sufficient information for correct staging and that various other staging systems are utilized to classify different types of cancer. Additionally, the potential influence of cancer related treatments on body composition and/or weight loss was not directly accounted for, although our patient groups were matched for cancer type and/or stage and therefore likely to have had similar treatments. Further, causation cannot be established due to the observational nature of our study. We acknowledge that larger, longitudinal, prospective cohorts are needed to expand upon our observations, but our study meaningfully contributes as hypothesis generating. Studies with extended follow-up may be able to fully elucidate the weight loss trajectory and long-term survival after cancer diagnosis.

## Conclusion

In conclusion, we report that presence of detectable BAT on ^18^F-FDG-PET-CT in treatment-naïve patients with cancer is associated with reduced weight loss (∼3.6%) and may lower the risk for developing cancer cachexia by 44% for up to a year after cancer diagnosis. These results support that detectable levels of BAT at diagnosis may result in a clinically significant reduction in the risk for developing cancer cachexia. The mechanism underlying the reported relationship is unclear. Further research is needed to clarify whether BAT is causally involved in the pathophysiology of cancer cachexia or whether it functions as a biomarker of improved cardiometabolic health or other (patho)physiological processes conferring an advantage in patients diagnosed with malignancies of various origins.

## Figures and Tables

**Figure 1:**
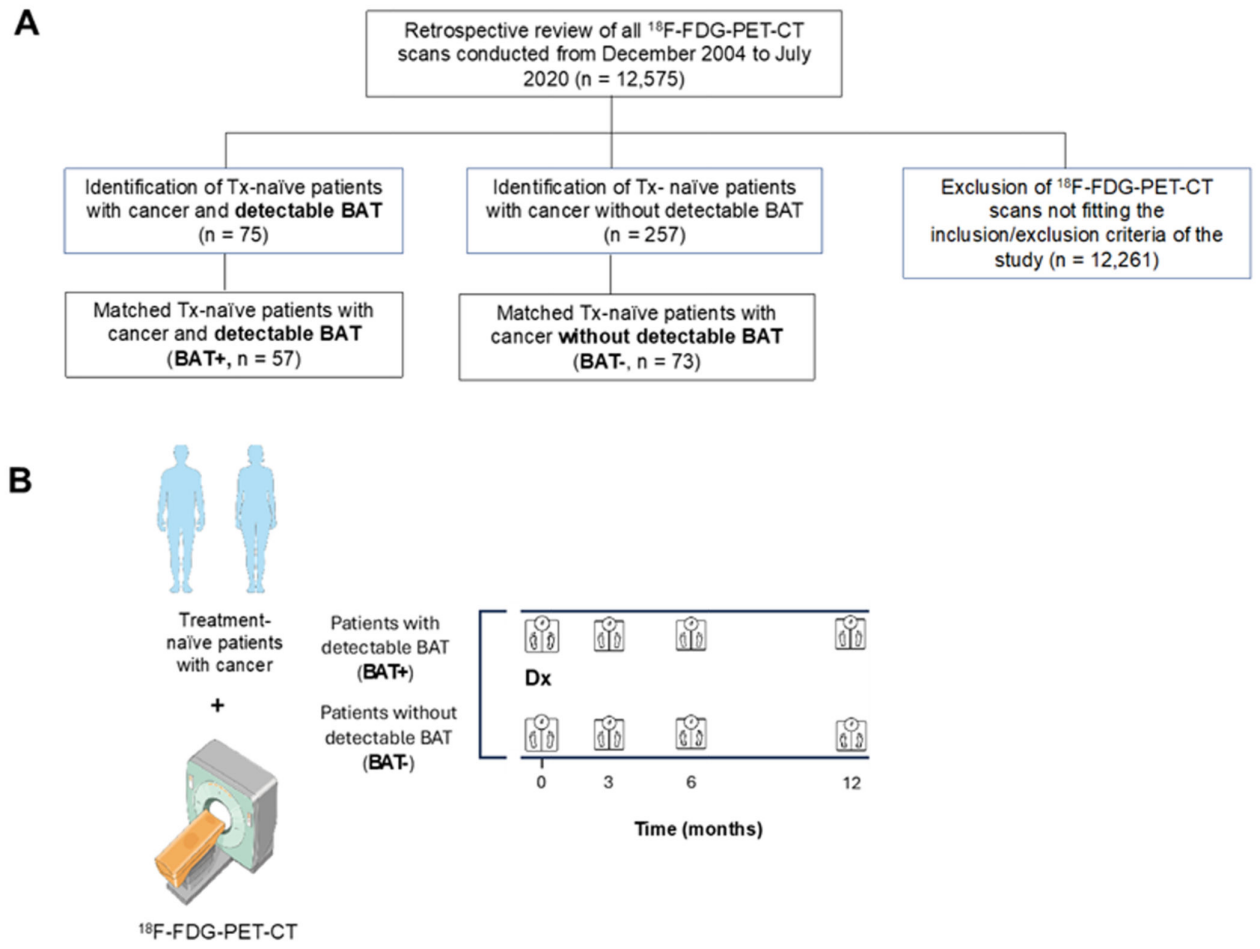
Overview of cohort selection (A) and study design (B). *Abbreviations: BAT, brown adipose tissue; BAT+, patients with detectable BAT; BAT-, patients without detectable BAT; Dx: diagnosis;* ^*18*^*F*^*-*^*FDG, 2-deoxy-2-[18F]fluoro-D-glucose; PET-CT: positron emission tomography-computed tomography; Tx: treatment*. Parts of the figure were drawn by using pictures from Servier Medical Art. Servier Medical Art by Servier is licensed under a Creative Commons Attribution 3.0 Unported License (https://creativecommons.org/licenses/by/3.0/).

**Table 1:** Demographic and clinical characteristics of the study patients. Data are mean ± SD for normally distributed data or median (interquartile range) for non-normally distributed data. Categorical variables are absolute and relative frequencies. *Abbreviations: BAT, brown adipose tissue; BAT+, patients with detectable BAT; BAT-, patients without detectable BAT; NA, not available; BMI, Body mass index, PET/CT: positron emission tomography -computed tomography*

Parameters	BAT- (n = 73)	BAT+ (n = 57)
**Age** (years)	58.5 ± 15.6	55.3 ± 18.0
**Sex, % women**	75.3	77.2
**Cancer Type**		
Breast	4 (5.5%)	4 (7.0%)
Gastrointestinal	0 (0%)	3 (5.3%)
Genitourinary	2 (2.7%)	6 (10.5%)
Head and neck	3 (4.1%)	2 (3.5%)
Hematologic	20 (27.4%)	15 (26.3%)
Respiratory	25 (34.2%)	15 (26.3%)
Skin	16 (21.9%)	9 (15.8%)
Other Cancers	3 (4.1%)	3 (5.3%)
**Cancer Stage**		
Stage 1	15 (20.5%)	10 (17.5%)
Stage 2	9 (12.3%)	14 (24.6%)
Stage 3	17 (23.3%)	12 (21.1%)
Stage 4	14 (19.2%)	6 (10.5%)
Unknown/NA	18 (24.7%)	15 (26.3%)
**Race**		
White	55 (75.3%)	48 (84.2%)
African American or Black	5 (6.8%)	2 (3.5%)
Asian	6 (8.2%)	3 (5.3%)
Other/Unknown	9 (12.3%)	4 (7.0%)
**Ethnicity**		
Non-Hispanic or Latino	60 (82.2%)	45 (78.9%)
Hispanic or Latino	5 (6.8%)	8 (14.0%)
Unknown	8 (11.0%)	4 (7.1%)
**Weight at diagnosis (kg)**	72.4 (61.2,86.6)	68.1 (59.5, 83.1)
**Height (m)**	1.69 ± 0.08	1.66 ± 0.09
**BMI at diagnosis (kg/m^2^)** **Temperature during day of** **PET/CT imaging (°C)**	25.3 (22.1, 29.7)	24.5 (21.5, 29.4)
Average	17.4 ± 6.8	15.4 ± 6.2
Minimum	9.9 ± 5.5	8.6 ± 5.1
Maximum	23.9 ± 8.6	22.0 ± 7.9
**Season of PET/CT imaging**		
Winter	25 (34.2%)	22 (38.6%)
Spring	21 (28.8%)	15 (26.3%)
Summer	19 (26.0%)	6 (10.5%)
Fall	8 (11.0%)	14 (24.6%)
